# Ultrasound-Guided Erector Spinae Plane Block in the Emergency Department: A Treatment for Intractable Sciatica

**DOI:** 10.7759/cureus.75932

**Published:** 2024-12-18

**Authors:** Carlos A Gonzalez-Cobos, Miguel J Rosa Carrasquillo, Miguel F Agrait Gonzalez

**Affiliations:** 1 Emergency Medicine, Hospital Episcopal San Lucas, Ponce, PRI; 2 Emergency Medicine, Ponce Health Sciences University, Ponce, PRI

**Keywords:** esp block, low back pain (lbp), lower extremity radiculopathy, sciatica pain, ultrasound-guided

## Abstract

Sciatica, often characterized by low back pain (LBP) radiating to the leg, is a challenging condition to manage, especially when conventional therapies fail. We present the case of a 27-year-old man who suffered from persistent low back pain with left-sided radicular symptoms. Despite treatment with numerous oral medications, including acetaminophen, nonsteroidal anti-inflammatory drugs (NSAIDs), gabapentinoids, and muscle relaxants, his symptoms persisted and intensified. Upon presenting to the emergency department (ED) for pain control, he underwent an ultrasound-guided erector spinae plane block (ESPB). The patient reported significant pain relief and functional improvement following the procedure. This case underscores the potential utility of the erector spinae plane (ESP) block as a safe and effective interventional approach for managing acute or chronic low back pain with radiculopathy in the emergency setting, particularly for patients unresponsive to conventional therapies.

## Introduction

Low back pain (LBP) remains one of the most prevalent complaints in emergency medicine, accounting for an estimated 4.2 million visits annually in the United States [1,2]. When accompanied by pain radiating down the leg, often referred to as sciatica or radiculopathy, it is frequently associated with nerve root compression due to herniated discs, spinal stenosis, or other lumbar spine pathologies. The condition poses significant challenges, as traditional therapies such as physical therapy, nonsteroidal anti-inflammatory drugs (NSAIDs), and neuropathic pain medications such as gabapentin often fail to provide complete relief.

Regional anesthesia techniques, including the erector spinae plane block (ESPB), have gained attention in recent years [3,4]. Initially developed for thoracic neuropathic pain, the ESPB has shown promise in treating acute and chronic spinal pain, although its application in managing sciatica remains less well-documented [5]. This case report highlights the successful use of an erector spinae plane (ESP) block for intractable radicular pain, showcasing its potential role as an adjunct or alternative treatment in the emergency department (ED).

## Case presentation

Case report

A 27-year-old man with no past medical or surgical history presented to the emergency department with worsening low back pain radiating down to his left heel over the past month. The patient described it as a sharp and burning sensation radiating down the lateral aspect of his thigh all the way down to the lateral aspect of his foot. His symptoms had been present for six months but had recently intensified despite adherence to a multimodal oral medication regimen. At the time of evaluation, he had no position that could provide comfort, and any movement would worsen the symptoms. He reported severe pain, scoring 9/10 on the visual analog scale (VAS), which limited his ability to perform daily activities, including walking, and necessitated his arrival in the ED by wheelchair.

The patient had previously been treated with a combination of ibuprofen 400 mg three times a day (TID), gabapentin 100 mg TID, and cyclobenzaprine 10 mg at bedtime, alongside several months of physical therapy. Despite these interventions, he reported no significant improvement. He denied any history of trauma, prior surgeries, or substance abuse and had no prior episodes of severe back pain before this onset. He also reported that he was scheduled to undergo a lumbar spine MRI without contrast but was still awaiting the appointment date for the study.

Physical examination revealed marked tenderness over the lower lumbar spine, particularly in the left paraspinal region. No step-off was identified. Neurological examination demonstrated mild sensory deficits consistent with L5-S1 radiculopathy, including hypesthesia along the corresponding dermatomes. Motor strength was grossly preserved with the exception of reduced strength on eversion in the left foot compared to the right foot. Patellar and Achilles deep tendon reflexes were normal bilaterally. Both straight leg raise and slump tests were positive for radicular pain on the left side. There was no evidence of saddle anesthesia or urinary or bowel incontinence. Lumbar spine X-rays showed no significant degenerative changes or structural abnormalities.

Given his refractory pain and clinical findings, the patient was counseled regarding the risks and benefits of an ESP block. After informed consent was obtained, the procedure was performed using ultrasound guidance.

Procedure

**Figure 1:**
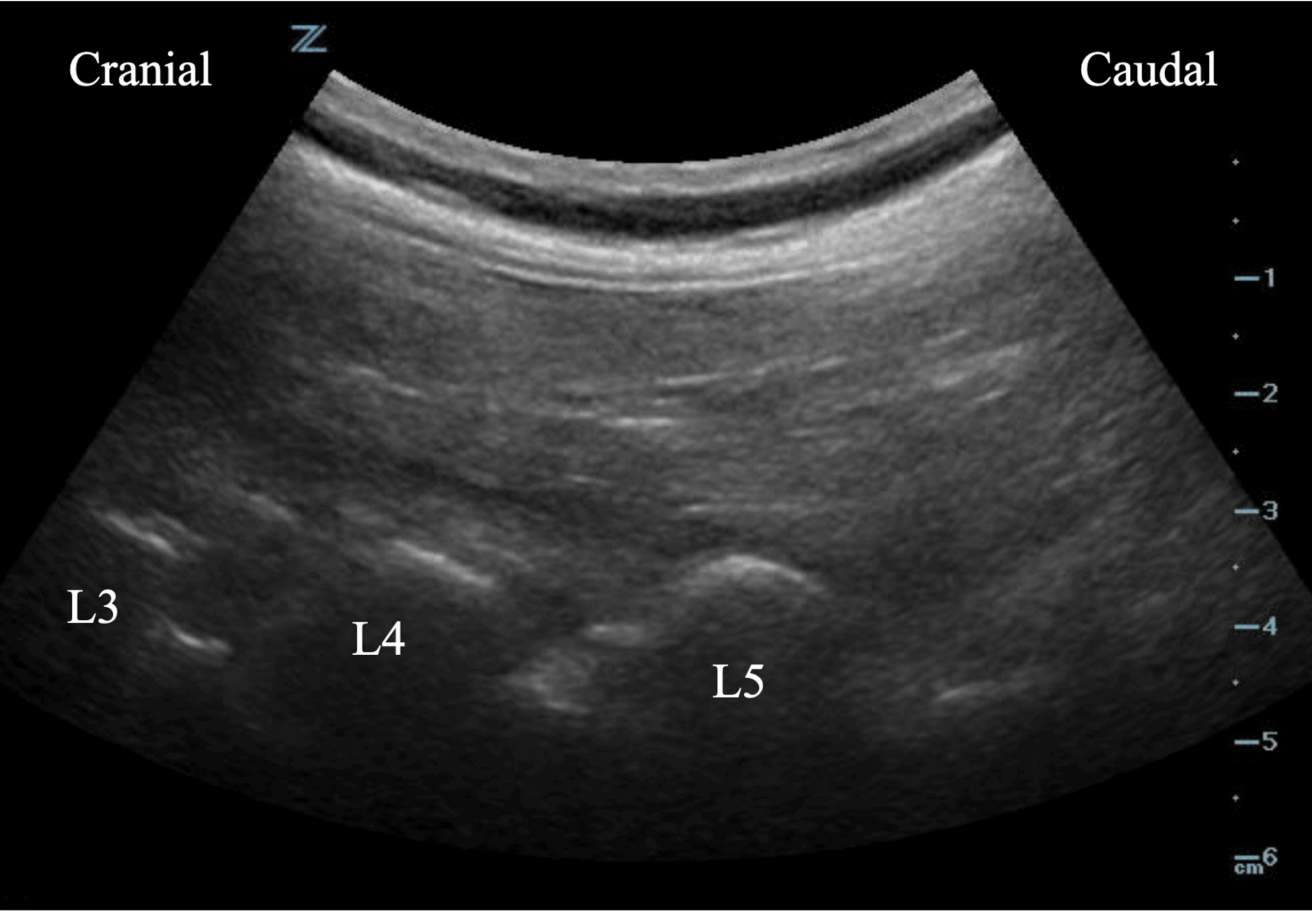
Image of the curvilinear transducer demonstrating lumbar transverse processes in the longitudinal plane L3, transverse process of third lumbar vertebrae; L4, transverse process of fourth lumbar vertebrae; L5, transverse process of fifth lumbar vertebrae

**Figure 2:**
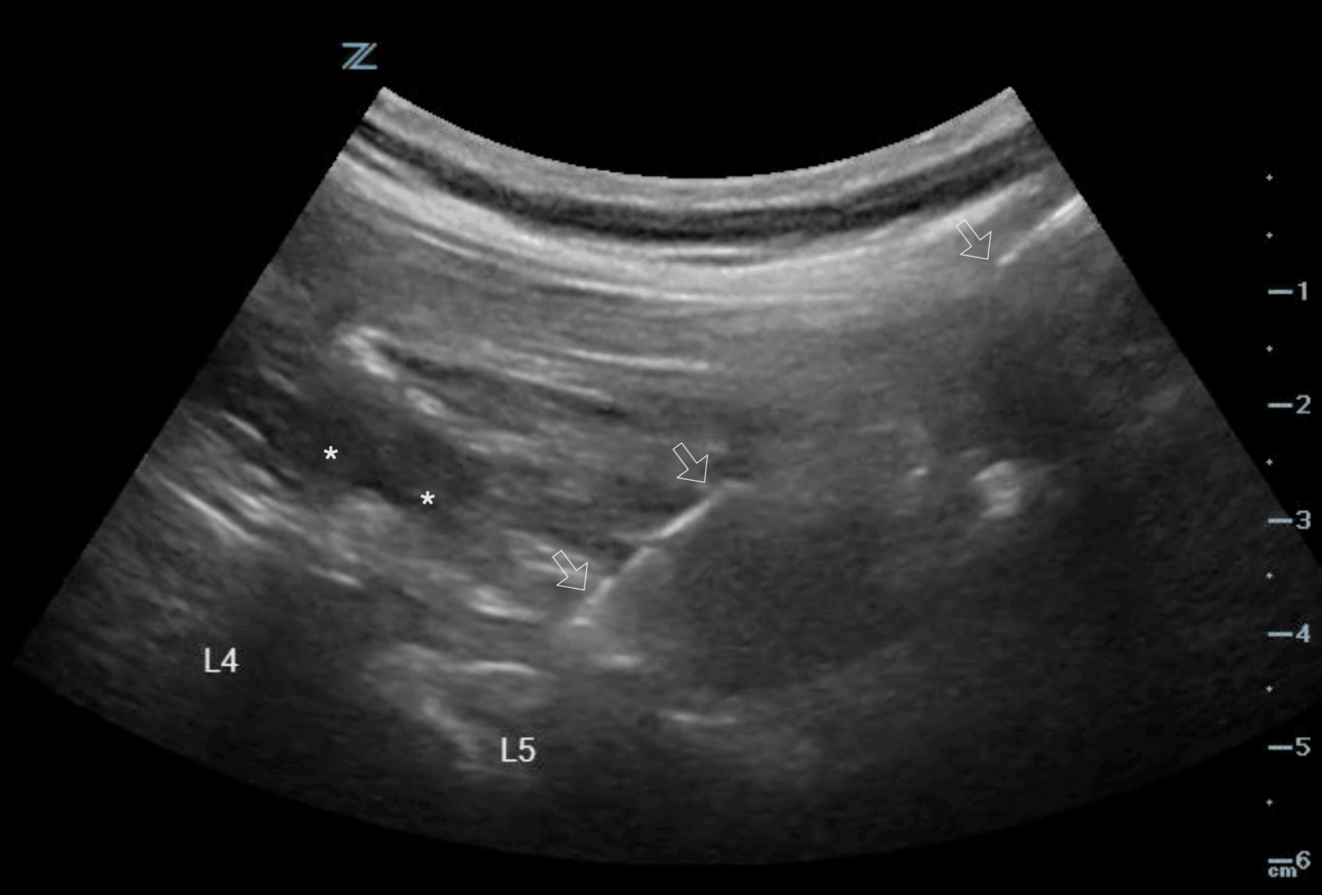
Image of the spinal needle inserted at the level of the ESP of the TP of L5 with a certain degree of hydrodissection L4, transverse process (TP) of fourth lumbar vertebrae; L5, transverse process of fifth lumbar vertebrae; arrows, path of the needle *Hydrodissection ESP: erector spinae plane

The patient was positioned prone on the stretcher. Cardiac monitoring was started, and following the sterile preparation and local infiltration of lidocaine, a 22-gauge, 3.5-inch spinal needle was advanced using an in-plane approach to the level of the L3-L4 transverse processes (TP) (Figure 1). With continuous ultrasound visualization, the needle was directed to the fascial plane of the erector spinae muscle at the level of the ESP on top of the L5 TP (Figure 2). After negative aspiration, a mixture of 20 mL sterile saline, 10 mL 0.5% bupivacaine, and 4 mg dexamethasone as an adjuvant was administered incrementally with visualized hydrodissection of the erector spinae plane. The procedure was well-tolerated without immediate complications.

Outcome and follow-up

After 30 minutes of observation and monitoring after the procedure, the patient reported significant pain reduction with a VAS of 3/10, allowing him to ambulate with minimal discomfort. He was discharged home in stable condition with instructions for follow-up care. At a one-week follow-up call, the patient described a dramatic improvement, with his pain reduced to 2/10 on the VAS. His leg symptoms, including numbness and tingling, had nearly resolved, and he had resumed daily activities, including light exercise. Under medical guidance, he successfully tapered off gabapentin and muscle relaxants.

A lumbar spine MRI without contrast performed post-discharge revealed mild disc desiccation and a 3 mm left foraminal disc herniation at L5-S1 causing moderate foraminal narrowing. He was advised to continue physical therapy focusing on core stabilization and to follow up with a multidisciplinary team, including pain management and spinal surgery specialists.

## Discussion

The management of chronic LBP with radicular symptoms normally necessitates a multidisciplinary conservative approach [5]. Treatment typically involves pain management strategies, physical therapy to improve mobility and strengthen supportive musculature, and, in some cases, steroid injections. For these cases, lumbar transforaminal epidural steroid injection would be the standard of care. However, patients require referrals, physical therapy, and appointments with a pain medicine specialist, which delays treatment and results in patients going to the ED for pain control. When conservative treatments fail, interventional techniques such as regional nerve blocks can offer effective pain relief [6]. Among these, the ESPB is emerging as a promising option for lumbar radiculopathy. Although initially developed for managing thoracic pain, the ESPB has shown utility in lumbar pain syndromes by targeting the dorsal rami of spinal nerves through the deposition of local anesthetics along the erector spinae muscle plane [7]. While its precise mechanism of action remains incompletely understood, it likely involves a combination of mechanical and neurophysiologic effects.

The ESPB has demonstrated its versatility in managing pain originating from multiple anatomical regions. In the ED, it has been successfully applied for rib fractures, renal colic, hepatobiliary pain, postherpetic neuralgia, and mechanical LBP with or without radiculopathy, among a few others [8-10]. These varied applications underscore its potential as a valuable analgesic tool. However, as with any interventional procedure, it carries inherent risks. Complications may include damage or infection to surrounding tissues, nerves, or vasculature, as well as symptoms such as numbness, weakness, or inadequate pain relief. Although rare, a notable risk specific to this procedure is local anesthetic systemic toxicity (LAST), which arises due to the vascular uptake of local anesthetic due to the high volumes required during plane blocks such as the ESPB. LAST can lead to severe neurological and cardiovascular complications, highlighting the importance of careful dosing, monitoring, and readiness to manage adverse effects. Additionally, contraindications include infection at the injection site, coagulopathy, significant anatomical deformities, or patient refusal.

Despite its growing popularity, the level of evidence supporting the ESPB remains limited in the ED setting. Current literature is predominantly composed of case reports, case series, and a few interventional studies. While pilot studies suggest promise in managing mechanical LBP and rib fractures in acute care, large-scale prospective randomized trials are lacking [11,12]. In contrast, its use in the anesthesia setting, where the technique was initially introduced, has been better studied, demonstrating its efficacy in perioperative pain control and reducing opioid requirements, as well as with prospective interventional studies proving its effectiveness [13].

In the case presented, the ESPB provided rapid and substantial pain relief, enabling the patient to regain functionality and avoid unnecessarily starting opioids. This outcome aligns with previous reports demonstrating its efficacy in both acute and chronic LBP. These findings highlight the potential of the ESPB as a valuable adjunct in the emergency setting. Notably, this procedure is not resource-intensive and typically requires the same materials as other nerve blocks commonly performed in the ED, with the exception of necessitating an assistant to inject the anesthetic during needle guidance. It can also be performed with various local anesthetics and adjuvants depending on department availability. The total time to complete the procedure is typically in the range of seven to 10 minutes, making it an efficient option for pain management in the emergency department. Although ESPB is not standard in the training of emergency medicine, it is a procedure that, depending on the training program, is being included as part of the armamentarium of procedures for pain management.

While the ESPB described in this case was performed using a longitudinal approach, it is important to note that multiple ultrasound-guided approaches exist for this procedure, expanding its versatility. These include the transverse approach in the supine position and the lateral decubitus position, often referred to as the “Aksu” approach. In terms of needle placement, variations include targeting the region just superior or inferior to the border of the transverse process, below the fascial plane, commonly referred to as the “Tulgar” approach. These alternative techniques offer flexibility in procedural execution and can be tailored to the patient’s anatomy and clinical context [14].

Future research should focus on defining optimal patient selection criteria, refining procedural techniques, and establishing the role of the ESPB within standardized pain management protocols and the time to discharge after the intervention compared to standard therapy. Additionally, further high-quality studies are necessary to validate its efficacy, particularly in the emergency medicine context, to strengthen the evidence base for this promising intervention.

## Conclusions

The erector spinae plane block is a promising interventional technique for managing refractory sciatica in the emergency department. This case highlights its effectiveness in providing rapid pain relief, improving functionality, and reducing reliance on systemic medications. As evidence grows, the ESP block may become a standard option for emergency physicians managing challenging cases of lumbar radicular pain.
